# The use and value of maps in Community-Oriented Primary Care: Does process matter?

**DOI:** 10.4102/phcfm.v12i1.2099

**Published:** 2020-02-05

**Authors:** Nina M. Honiball, Tessa S. Marcus

**Affiliations:** 1 School of the Arts, Faculty of Humanities, University of Pretoria, Pretoria, South Africa; 2 Department of Family Medicine, School of Medicine, Faculty of Health Sciences, University of Pretoria, Pretoria, South Africa

**Keywords:** Maps, Mapmaking, Community-Oriented Primary Care, Ward-Based Outreach Teams, Healthcare Delivery

## Abstract

**Background:**

Maps are important tools in healthcare delivery. In Community-Oriented Primary Care (COPC), they are expected to be used to plan services and resources for defined geographical areas, delineate team practice areas, allocate healthcare workers to households and support service delivery and performance management.

**Aim:**

This is a study of the use and value of maps and mapmaking in the delivery of healthcare services through Ward-Based Outreach Teams (WBOTs).

**Setting:**

This study was conducted between 2014 and 2016 in Mamelodi (South Africa), an urban settlement selected to begin the City of Tshwane’s WBOT implementation programme in 2013.

**Methods:**

This study is based on three qualitative participatory mapmaking projects with WBOT healthcare professionals and workers. Data generated through mapmaking, focused group discussions, individual semi-structured interviews, reflective writing and feedback workshops were analysed thematically.

**Results:**

Through mapmaking and discussions about the maps, healthcare providers took ownership of the maps they were creating or viewing, added their own information onto the maps, voiced issues about their practice, generated new knowledge and shared ideas and solutions for challenges. These processes expanded the use and value of maps beyond assisting participants to gain insights into the context, people and organisations of their places of work.

**Conclusion:**

Maps become creative learning tools that can be used in emergent ways to solve healthcare service and other problems when they are actively generated and engaged through facilitated discussion and reflection. This allows WBOTs to see maps as dynamic canvasses that they can use to improve service delivery.

## Introduction

Maps are important tools in healthcare delivery. In public health, medical data maps generated with Geographic Information Systems (GIS) are commonly used to study population health in the context of space and place.^1^ Among other things, they help to identify populations at risk and the environment where people live or work, the relationship between health outcomes and risk factors in those environments, and to plan healthcare-related interventions.^1^ In clinical care, maps are used to monitor and evaluate healthcare services,^2,3,4,5^ to assist with planning and implementation^4,6^ and to enable community engagement in healthcare delivery.^5,7,8,9^

Maps are elemental to the design, planning and implementation of Community-Oriented Primary Care (COPC). Following the initiation of primary care re-engineering in 2010 by the National Department of Health, the University of Pretoria at the Department of Family Medicine (UPDFM) partnered with Gauteng Province (2010–2012) and the City of Tshwane (2013–2016) to implement COPC in the Tshwane District through Ward-Based Outreach Teams (WBOTs). As an approach to health service delivery that combines the tenets of public health and clinical care,^10^ COPC gives importance to place and space^11^ in both health and healthcare service delivery as it seeks to render quality, person- and family-centred, generalist healthcare to people in defined geographic communities.

Maps are used in four ways in the contemporary iteration of COPC. Google Earth maps are used as a key element in planning. In the Integrated Health Planning System (iHPS), toolkit designed and developed by the UPDFM, maps are generated to visualise the best available demographic, socio-economic, health and service data in order to support macro- and middle-level healthcare resource allocation to geospatially defined communities.^12^ Cadastral maps, as general land administration tools, are used to allocate community-based teams and individual Community Health Workers (CHWs) to defined geographic areas and households. Community health workers also create hand-drawn sketch maps to help them identify their areas of practice. These are used on their own or when available to complement cadastral maps. Lastly, GIS and mapping components are built into AitaHealth™, a customised application device and web Information and Communication Technology (ICT) platform, developed by UPDFM and Mezzanineware (Vodacom). The platform generates maps from data collected by CHWs on mobile handsets during household registration, screening and service delivery. These maps support service prioritisation, capacity development and performance management by visualising micro-level household and individual health status.

Conceptually, maps are what Kitchin and Dodge term sets of spatial practices that people use to solve ‘relational problems’.^13^ Although maps are often thought of as static, ontologically secure representations of reality, they are in fact momentary transitory objects produced and recreated by people through use.^13^ Corner also argues that maps trigger agency because they enable people to generate ideas and make connections between layers of information that are visualised on a map.^14^

This article presents the results of a study of maps and mapmaking with healthcare professionals and CHWs to better understand their use and value in the delivery of healthcare services through WBOTs.

## Methods

### Study design

The study involved three participatory mapmaking projects. The Local Institutional Support Assessment (LISA) project was initiated around a collection of existing maps drawn by CHWs and their use of the LISA tool to identify and determine possible organisational support for COPC in their practice communities. The ‘history of health’ project explored personal experience of the local geography of healthcare during the 1980s under apartheid and its relevance for contemporary healthcare. Finally, the ‘community health’ project explored Tuberculosis (TB) incidence and household size using data collected on AitaHealth™ by CHWs in their practice communities. The maps in this project were generated by QlikView software.

### Setting, study population and sampling

The study was conducted in Mamelodi, the geographic area selected by the Tshwane metropolitan municipality in 2013 to begin a citywide phased implementation of WBOTs. During 2014–2016, 22 teams and approximately 321 CHWs working there represented the most extensive and sustained application of COPC in any ward in Tshwane and across the country.

The study population included all healthcare practitioners involved in COPC who routinely worked in WBOTs in 12 of 14 wards in Mamelodi and Nellmapius. Participants included retired professional nurses, each of whom served as a team leader to 12 or more CHWs and Family Medicine registrars.

All teams were informed about the purpose of the study and invited to take part in the study during a monthly team leader meeting. Participants were selected using purposive sampling. Inclusion criteria depended on the requirements of the mapmaking project and individual interest and availability. Only CHWs were involved in the ‘LISA’ project. Only professional nurses were involved in the ‘history of health’ project. And a mix of professional nurses, CHWs and doctors were involved in the ‘community health’ project (Table 1). All the research was conducted in English. Where participants spoke in Sepedi, their inputs were translated by a Sepedi-speaking research assistant during the sessions or through transcription.

**TABLE 1:** Study participants by map project.

Map project	Participants
LISA	45 CHWs
History of health	19 Professional nurses
Community health	7 Professional nurses
	7 CHWs
	7 doctors

### Data collection

All three participatory mapmaking projects followed a common process, namely, a mapmaking activity, a focused group discussion about the activity, individual reflective writing and a results feedback workshop 1 month after data collection. In addition, in the ‘community health’ project, individual semi-structured interviews and small group discussions were conducted with a team leader, a CHW and a registrar linked to each team around a map of TB in their designated area of service.

**FIGURE 1:**
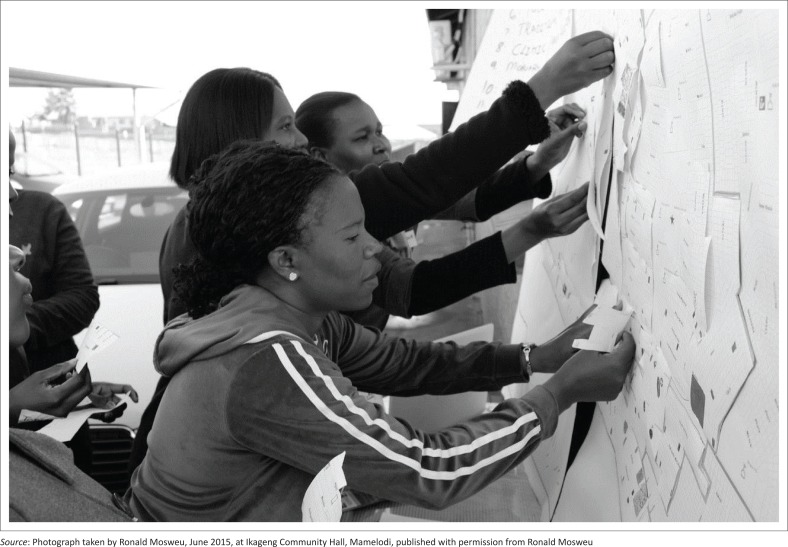
Community health workers adding map layers onto the ‘LISA’ map

**FIGURE 2:**
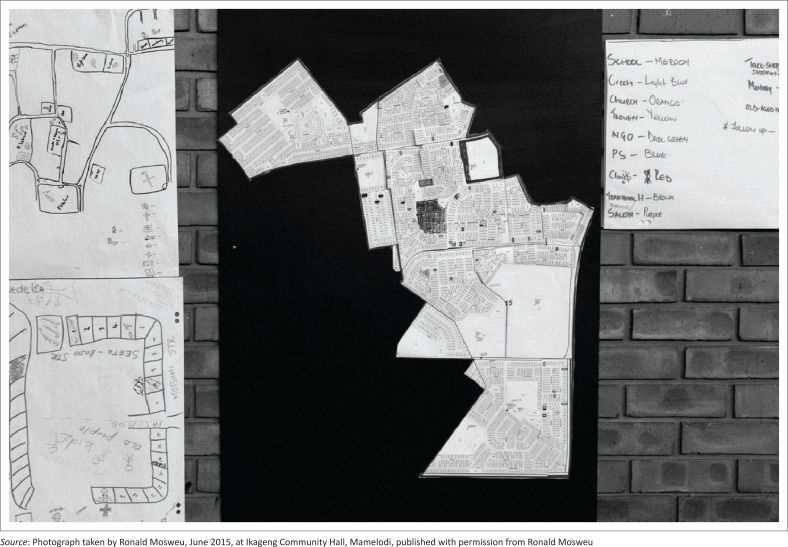
Example of a ‘LISA’ map – The image shows the first composite layer put together by participants which visualises the type of stakeholders in an area.

The ‘LISA’ project visualised routine organisational data on local institutional services collected by CHWs on paper. Using three copies of a town planning map (Figure 1), the participants identified (1) the types of organisations in their ward (layer 1), (2) the willingness of each organisation to take part in COPC (layer 2) and (3) CHW contact with organisations (layer 3). Community health workers used their own colour coding system in the first layer (Figure 2). Green, orange or red was used in the second layer to show degrees of organisational interest in COPC. For the third layer, CHWs’ contact with organisations was depicted in blue (no contact) and purple (contact).

**FIGURE 3:**
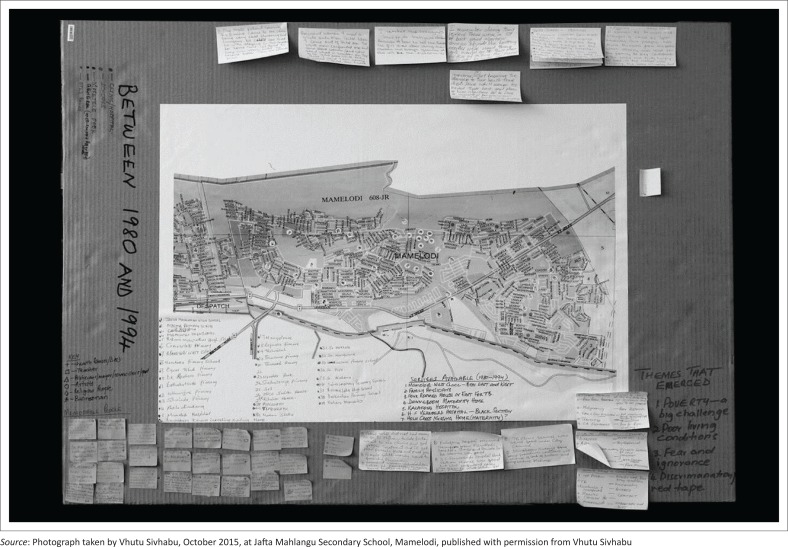
Example of a ‘History of Health’ project map generated by a group of participants.

The ‘history of health’ project drew on the participant’s personal experience. Through participatory mapping, they identified three topics, namely, (1) significant landmarks and social spaces as well as their home addresses in the 1980s; (2) key healthcare providers and significant individuals who contributed to local health and healthcare delivery; and (3) common conditions and illnesses. Using coloured pens, posted notes and sticky dots, they worked in groups, creating their own systems to log, add and categorise the information they generated on their maps. Each topic was mapped onto a 1991, A1-sized roadmap of Mamelodi that was printed and mounted on sturdy cardboard (Figure 3).

**FIGURE 4:**
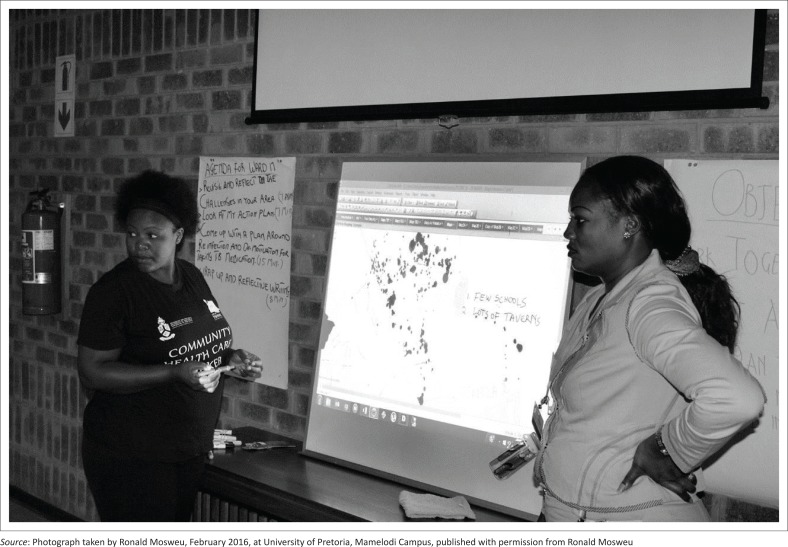
Community health workers and registrar doctor drawing on a map projection in the ‘Community Health’ project.

Data for the ‘community health’ project were collected by CHWs on AitaHealth^TM^ as part of routine service delivery. Participants were grouped according to their specific areas of practice and shown five local ‘household size and TB incidence’ data maps. Data on each were organised by subject and visualised by coloured dots on symbol maps (Figure 4) *inter alia*, Map 1 – population density (dark blue); Map 2 – households with diagnosed TB (green), Map 3 – households where TB had been diagnosed (green) and those where there was possible but undiagnosed TB (orange); Map 4 – households where one or more household members were diagnosed with TB but not on treatment (red); and Map 5 – households with members both diagnosed but not on treatment (red) and possible TB but undiagnosed (orange). Each map was explored through guided individual interviews and in focused group discussions.

The three mapping projects were repeated three times with groups of 4–12 participants to ensure data saturation.^15^ Fieldwork was undertaken over a 12-month period between 2015 and 2016.

The questions guiding the discussion in the focus groups were informed by visual rhetoric, a subject area that dates back to Aristotle and Ancient Greece.^16^ Maps are examples of visual rhetoric because, as images, they give preference to the display of certain types of information and are constructed with specific meanings in mind.^17^ Using Foss’s framework to identify the ‘nature of the visual rhetoric’ in an image, participants were asked to describe and discuss both the presented and suggested elements that they saw in each map.^18^ This involved them looking for and naming all the elements they saw – such as colour, shape, form and texture – and then exploring ideas, themes, insights and concepts that could be deduced from an image.

### Data analysis

Focus group discussions and in-depth interviews were audio-recorded, translated and transcribed verbatim. Individual reflective writing and session evaluation forms were digitised. Data were analysed thematically, following the six-phase process described by Braun and Clark^19^ and supported by NVivo.

### Trustworthiness

Credibility, transferability, dependability and confirmability were used as criteria to evaluate the trustworthiness of the methodology.^20^ Fairness, ontological authenticity and educative authenticity were used to evaluate authenticity in the mapmaking process.^20^

Credibility was ensured through formal and informal member checking^20^ throughout all mapmaking processes. Transferability was attained by providing a ‘thick description’^20^ of the processes and the conditions needed to implement each one of the three mapmaking projects. Dependability was evaluated by documenting the way in which the mapmaking processes were implemented in practice. Confirmability was achieved by making the data analysis process transparent and available for independent review.

Fairness was achieved through open negotiation of participants’ views in both focus group discussions and data analysis presentations. Ontological authenticity was determined by comparing participants’ initial and final written articulations of what maps meant to them. Educative authenticity was enabled through the sharing of ideas in both focus group discussions and data analysis presentations.

### Ethical considerations

Ethical clearance was obtained from the Research Ethics Committee of the Faculty of Health Sciences, University of Pretoria (clearance number: 160/2015). Participants gave informed consent in writing. In the third project, community data maps generated with QlikView software displayed locally relevant, household-specific data to their related service provider teams to support micro-service delivery. Otherwise, the information presented by the researcher to groups or in presentations was anonymised.

## Results

The ‘LISA’ project process assisted CHWs to evaluate their work and plan further engagements with these organisations and institutions:

By using these three layers, we can see where we are and where we can re-correct us by giving health talks or explaining to the people about COPC. And, to check the gaps from our map. We also get challenges from the community especially the stakeholders like traditional healers and tavern owners. (MP1–P43, CHW, female, Ward 16, reflective writing)

The three layers can help us to know our area very well, and the three colours will tell us whether we are working well or we are not working well. (MP1–P41, CHW, male, Ward 16, reflective writing)

… [*The map*] helped us [*to*] be aware of the people, businesses, and organisations we haven’t reached. If we use colours and different layers it would help us reach our monthly target. (MP1–P24, CHW, female, Ward 15, reflective writing)

Through discussions about the information visualised on the map, participants formulated insights and generated new knowledge:

… [*A*]t Ward 93, there’s lots of tuck shops and taverns, lack of road infrastructure overcrowding and limited open space also dumping [*illegal rubbish*] is the main challenge in this area. NB Only one creche for the whole area and it [*the map*] shows unemployment and unhealthy eating; it shows us the people who are not part of WBOT, some are scared about license [*shops trading illegally*]. And there’s no space for recreation (sports ground). (MP1–P21, CHW, female, Ward 15, reflective writing)

According to these layers, especially layer 1, we don’t have enough space where they can build a high school and another clinic. Learners move from ward 16 to other wards, and we have problems with overcrowding at our clinic… It is a good thing that we have a lot of spaza shop because we are able to get fresh bread and other things that we need. So, it’s unfortunate that we have lots of taverns. When people get drunk, they can practice unsafe sex. [*Tavern*] owners need to practice health talks and [*have*] health promotion pamphlet at their places. (MP1–P39, CHW, female, Ward 16, reflective writing)

The project also gave participants a chance to identify gaps in awareness about and the need to address issues of trust in COPC among organisations:

… [*A*]ccording to the [*second*] layer presented to us there are lots of green colours that really show us that some of the organisations are interested to work with us and for those who refused to speak with us, it shows that they were unclear about what COPC is and what we are doing. (MP1–P39, CHW, female, Ward 16, reflective writing)

The second layer gives us input that too many of our organisations don’t have trust in our work. And at least we have some that are in, but we still need to work more on getting others. (MP1–G2, CHW, female Ward 15, focus group discussion)

The ‘history of health’ project enabled participants to look into the past as a group. This gave participants a different appreciation of their colleagues and their community context:

I enjoyed working with others because in the end we became one family and we were complementing each other, reminding each other of certain events that some could highlight better than others. (MP2–P7, team leader, female, Ward 97, reflective writing)

(The workshop) enabled us to share ideas and know our community and surroundings better. (MP2–P1, team leader, female, Ward 28, reflective writing)

Learnt a lot from getting different views from colleagues and hearing how people interviewed feel and see [*the*] same issues differently. (MP2–P2, team leader Ward 28, female, reflective writing)

What I liked was learning about people who contributed to the community in various areas – health, education, business, community involvement, etc. One gained, as I was out of Mamelodi most of my adult life. Learning about the segregation at certain health facilities and seeing where we are today. (MP2–P3, cluster manager, female, reflective writing)

The process allowed participants to consider both negative and positive differences between present and past healthcare services:

Clinics were not as overcrowded, and people were not turned back. Medicines were never out of stock and clients received the best medications. (MP2–P2, team leader, female, Ward 28, reflective writing)

People are still using the bucket system for sewerage disposal in informal settlements. There is a need to build a new hospital in the East of Mamelodi as there are more people and mostly young people in the East in comparison to the West of Mamelodi. A second clinic is also necessary to be built in the West of Mamelodi to relieve overcrowding of clients in the existing Mamelodi West Clinic. (MP2–P4, team leader, female, Ward 93 West, reflective writing)

In comparison with the 1980s, there is an improvement with health issues, i.e. more clinics have been built. [*The*] Community is getting information, which means a reduction of diseases. (MP2–P6, team leader, female, Ward 17, reflective writing)

They’re getting [*healthcare*] education. There was no education. People are getting education now. Children are being immunised. (MP2–D1, Group 1, female, group discussion)

By visualising participant data, the ‘community health’ project brought the data to life:

Looking at the table, it’s just names there that shows you so many people [*have TB*]. But looking at the map, it shows you where actually that person is so that you can concentrate on that area… It actually gives you your ward as it is and you know that it means I must go to this area where the problem is. (MP3–P2, team leader, female, Ward 86, Team 2)

Maps generated helped participants see how to prioritise their actions and plan service delivery:

The maps generated are very useful because they give me feedback of what I am doing in the community through the CHWs. They also show me which action to take next from the maps that I saw. (MP3–P15, registrar, female, Ward 16)

I can see there are certain households who are having too many problems, so I must put more effort and should be concerned about those households. (MP3–P11, CHW, female, Ward 40)

They also helped them to monitor and evaluate their performance and progress:

The data is more clear to me because it shows how much work I have done so far. (MP3–P9, CHW, female, Ward 86, Team 2, reflective writing data analysis presentation)

I liked that TB cases are not too much in my area – because we always campaign about it and teach them [*community members*] that TB is curable and how to prevent it. (MP3–P13, CHW, female, Ward 86, Team 3, reflective writing)

We will use these types of maps to indicate the disease we have captured and, also to determine the difference we make in the community. And we can also use these maps to see how people are improving on their medication and how many people still need us to put more effort into them. (MP3–P11, CHW, female, Ward 40, reflective writing)

And they motivated them because they could see the value of their work:

The data we captured is a clear picture of our community, and we can see the accuracy and the value of our job. (MP3–P9, CHW, female, Ward 86, Team 2, reflective writing)

In a way, it encourages you that at least you’ve come this far. (MP3–P2, team leader, female, Ward 86, Team 2, interview)

I think the visual will give a sense that it’s something that’s doable. (MP3–P19, registrar, male, Ward 40, interview)

Group discussions around the data displayed on each respective team’s map prompted participants to explore discrepancies or anomalies in what they ‘knew’ and the information they saw:

I’m surprised because with the group that I am working with, they will always give me verbal reports that uh, no we found this HIV patient who is coughing a lot, signs and symptoms of TB are there, but the person is in denial, the person said we must not come back, does not want us in the house. Now how … so the map must correlate with verbal reports that they give me. (MP3–P6, team leader, female, Ward 40, Team 2, interview)

I mean just thinking of the number of people that come to the clinic with TB and then seeing how few people are captured with TB. There seems to be a discrepancy I would say. (MP3–P18, registrar, male, Ward 86, interview)

The maps also prompted discussions about the poor link between information collected in the community- and facility-based services:

I am not sure that the information I am collecting is going to benefit my community because I have collected data [*and*] no action has been taken. (MP3–P9, CHW, female, Ward 86, Team 2, reflective writing)

The community [*is*] asking us about our job. What are you doing with our information without giving answers to us? After doing registration, are you going to take us to the clinic & hospitals? They [*the clinic*] don’t take our referral clients seriously. (MP3–P10, CHW, female, Ward 28, reflective writing)

And they led to discussions about possible ways to improve cooperation between WBOTs and facilities as well as with partners in the community:

It might be good to present some of the data that we feel that’s relevant to the clinic. Just for them to understand what the data is about. I think they don’t understand it. (MP3–P18, registrar, male, Ward 86, interview)

I think one of the recommendations also is just for us to build a relationship with Stanza One [*Clinic*] so that they are aware of what we are doing in the community, because our job, and what the CHWs are doing, is alleviating the clinic work. (MP3–P15, registrar, female, Ward 16, group discussion)

Also, part of COPC is to involve the local politicians. I mean a map like this, you can show to the Ward Councilors … if you need to [do] an intervention … you involve them so that they can help with the security concerns … They’ve also got community meetings. So, actually, a map like this can [*also*] be shown at community meetings, so that people take ownership. (MP3–P19, registrar, male, Ward 40, interview)

## Discussion

Considering the results of this study of maps and mapmaking, we focus on four concepts, namely, purpose, emergence, method and agency.

Maps are generated and used to solve ‘relational problems’.^13^ A map’s purpose of use is, therefore, an important factor that determines its value to people. In the study, the purpose of the maps derived from their incorporation into the participatory mapmaking projects. By adding their own information and thoughts, participants could take ownership of the maps they were creating or viewing. This happened both through mapmaking and discussions about the maps. In the process, the map was emergent in nature,^13^ being ‘(re)made’^13^ by each group into something that was both useful and valuable to participants. Transformed in this way, maps and mapmaking are more likely to be used and adopted by people in their practice.

The three maps were made from a different medium. Each had a different lifespan, and although the projects were designed around the same process, they each included different activities. The ‘LISA’ project uses a physical mapmaking process where CHWs added information onto a paper-based map. This map was a once-off project, had a short lifespan and enabled CHWs to learn more about the environment where they worked in a tactile and visual way. In the ‘history of health’ project, participants generated their maps through participatory mapping. Similar to the way they are used in Participatory Rural Appraisal (PRA) projects, the ‘history of health’ project used participatory mapping as a tool to enable participants to take part in a learning process,^21^ work together and record their knowledge of a topic or place onto a physical map.^22^ The method is meant to create maps that are temporary in time and context specific in space. Here, although the medium was more amenable to repeated use, the outcome was that participants were able to work together, share untold historical information and experiences and learn from each other without the maps being readable or useful to anybody outside of the project itself.

Lastly, the ‘community health’ project used digital maps that were projected, analysed and discussed among teams of healthcare practitioners. These maps were created to display new or updated data collected by the participants or their teams. This made them useful for monitoring and improving services because they were formulated in real time and directly linked to service delivery. They were also more readable to others outside the project.

Through the three projects, the study exposed participants to the potential use and value of different types of maps for their practice. In the process, their perception of maps changed as they came to see them less as ready-made, once-off artefacts and more as tools that could and should be used repeatedly to resolve problems and improve service delivery. Corner^14^ calls this ‘the agency within mapping’. He argues that the map becomes a canvas on which people could map out, explore and contemplate new possibilities, ideas and insights. They draw on the insights revealed by and discovered in the process of creating to imagine and re-imagine different scenarios and realities.

The study also shows that incorporating maps into participatory mapmaking processes makes it possible to expand their use and value beyond the practices already described in the literature. In the mapmaking projects, three factors came together that enabled participants to discover the agentic^23^ potential of maps, namely, the maps themselves as visual artefacts, the participatory acts of mapmaking and group discussions of the maps. Through their actions and engagement with the maps, the participants gained insight into the context of their places of work, the people they serve, and the people and organisations they work with. They were also able to generate new knowledge, voice concerns about challenges and work together to resolve problems. The processes, therefore, took maps and mapmaking beyond their usual use and known value in planning, monitoring and evaluating service delivery.

## Limitations

Several factors limit the findings of this study. The three mapmaking projects were only implemented and tested with a small number of participants in one geographic area. The results are therefore not generalisable and require further testing at scale and across different sites. The transferability of the findings may also be limited because they are derived from qualitative research with WBOTs that implement ICT-enabled COPC and use curriculated workplace learning in their practice. As a cross-sectional study, the durability and long-term impact of the study results are not known. Lastly, the maps generated from the participatory mapmaking projects are not intended to be finished end products or artefacts. Rather, by design, they are works in progress, and as such, they are likely to yield different content and interpretations with each iteration.

## Recommendations

For maps to have use and value to healthcare providers at micro- and meso-levels, participatory mapmaking projects need to be an active part of primary healthcare service delivery, including the delivery of community-based services.

Further research is needed into participatory mapmaking in different settings and with different types of WBOTs. There is also a need for longitudinal research to determine the value and role of map use in COPC over time and the impact of maps and mapmaking on the provision and quality of healthcare.

## Conclusion

Working with maps in the three participatory mapmaking projects transformed participants’ perceptions about their use and value. When maps are actively generated and engaged, they become creative tools that can be used in emergent ways to solve healthcare service and other problems. This, in turn, allows WBOTs to see maps not as static objects, but as dynamic canvasses that they can use to help them in their practice. In this study, maps enabled healthcare providers who deliver community-based services in Mamelodi to simultaneously see and understand their areas of work, their practices and the challenges they faced. The processes used in the mapmaking projects helped them look for possible solutions, thereby transforming maps into tools to improve learning and stimulate agency in service.

## References

[1] Cromley EK, McLafferty SL GIS and public health. 2nd ed. New York: The Guilford Press; 2011.

[2] Hayashi AS, Bazemore A, McIntyre J Transforming community health and primary care education using clinical and administrative data and geographic information systems. JMap Geogr Libr. 2011;7(1):61–70. 10.1080/15420353.2011.534690

[3] Bazemore A, Diller P, Carrozza M The impact of a clinic move on vulnerable patients with chronic disease: A geographic information systems (GIS) analysis. J Am Board Fam Med. 2010;23(1):128–130. 10.3122/jabfm.2010.01.090103 20051553

[4] Dulin MF, Ludden TM, Tapp H, et al Geographic information systems (GIS) demonstrating primary care needs for a transitioning Hispanic community. J Am Board Fam Med. 2010;23(1):109–120. 10.3122/jabfm.2010.01.090136 20051550

[5] Hardt NS, Muhamed S, Das R, Estrella R, Roth J Neighborhood-level hot spot maps to inform delivery of primary care and allocation of social resources. Perm J. 2013;17(1):4–9. 10.7812/TPP/12-090 PMC3627788 23596361

[6] Lofters A, Gozdyra P, Lobb R Using geographic methods to inform cancer screening interventions for South Asians in Ontario, Canada. BMC Publ Health [serial online]. 2013 [cited March 2019];13(1):395 Available from: https://bmcpublichealth.biomedcentral.com/articles/10.1186/1471-2458-13-395 10.1186/1471-2458-13-395 PMC3640962 23622426

[7] Aronson RE, Wallis AB, O’Campo PJ, Schafer P Neighborhood mapping and evaluation: A methodology for participatory community health initiatives. Matern Child Health J. 2007;11(4):373–383. 10.1007/s10995-007-0184-5 17295067

[8] Beyer KM, Comstock S, Seagren R Disease maps as context for community mapping: A methodological approach for linking confidential health information with local geographical knowledge for community health research. J Community Health. 2010;35(6):635–644. 10.1007/s10900-010-9254-5 20352481

[9] Sage WM, Balthazar M, Kelder S, et al Mapping data shape community responses to childhood obesity. Health Aff (Millwood). 2010;29(3):498–502. 10.1377/hlthaff.2010.0153 20194992

[10] Gofin J, Gofin R Community-oriented primary care: A public health model in primary care. Rev. Panam. Salud Publica. 2007;21(2/3):177 10.1590/s1020-49892007000200012 17565804

[11] Marcus TS Community oriented primary care. Cape Town: Pearson Education South Africa Ltd; 2013.

[12] Bennett R, Marcus TS, Abbott G, Hugo JF Scaling community-based services in Gauteng, South Africa: A comparison of three workforce-planning scenarios. Afr J Prim Health Care Fam Med. 2018;10(1):1–7. 10.4102/phcfm.v10i1.1748 PMC6018689 29943603

[13] Kitchin R, Dodge M Rethinking maps. Prog Hum Geogr. 2007;31(3):331–344. 10.1177/0309132507077082

[14] Corner J The agency of mapping: Speculation, critique and invention In: Cosgrove D, editor Mappings, London: Reaktion Books; 1999, p. 213–252.

[15] Casey MA, Krueger RA Focus groups: A practical guide for applied research London, Thousand Oaks, and New Delhi: Sage; 2014.

[16] Foss SK Theory of visual rhetoric In: Smith KL, Moriarty S, Kenney K, Barbatsis G, editors Handbook of visual communication: Theory, methods, and media. London, New York: Routledge; 2004 p. 141–152.

[17] Kostelnick C Melting-pot ideology, modernist aesthetics, and the emergence of graphical conventions: The statistical atlases of the United States, 1874–1925 In: Hill CA, Helmers M, editors Defining visual rhetorics. New York: Lawrence Erlbaum Associates; 2004, p. 215–242.

[18] Foss SK Framing the study of visual rhetoric: Toward a transformation of rhetorical theory In: Hill CA, Helmers M, editors Defining visual rhetorics. New York: Lawrence Erlbaum Associates; 2004, p. 303–313.

[19] Braun V, Clarke V Using thematic analysis in psychology. Qual Res Psychol. 2006;3(2):77–101.

[20] Guba EG, Lincoln YS Fourth generation evaluation. London, Thousand Oaks, and New Delhi: Sage Publications; 1989.

[21] Chambers R Whose reality counts? Putting the first last London: Intermediate Technology Publications; 1997.

[22] Lydon M Finding our way home: Community mapping helps residents define their worries and realize their dreams. Altern J. 2000;26(4):26.

[23] Bandura A Social cognitive theory: An agentic perspective. Annu Rev Psychol. 2001;52(1):1–26. 10.1146/annurev.psych.52.1.1 11148297

